# Survey of Pancreatic Enzyme Replacement Therapy Dosing Experiences in Adults with Exocrine Pancreatic Insufficiency

**DOI:** 10.3390/healthcare11162316

**Published:** 2023-08-17

**Authors:** Dana M. Lewis, Arsalan Shahid

**Affiliations:** 1 OpenAPS, Seattle, WA 98101, USA; dana@openaps.org; 2 CeADAR—Ireland’s Centre for Applied AI, University College Dublin, D04 V2N9 Dublin, Ireland

**Keywords:** real-world evidence, pancreatic enzyme replacement therapy, EPI, PEI, exocrine pancreatic insufficiency, PERT

## Abstract

Objectives: Pancreatic enzyme replacement therapy (PERT) is essential for treating exocrine pancreatic insufficiency (EPI), a condition where the pancreas does not produce adequate enzymes for digestion. This study delves into the real-world experiences of individuals with EPI regarding their PERT usage. Methods: A study was executed using a tailored survey targeting individuals with EPI. Quantitative data analysis assessed factors such as age, duration of EPI, elastase levels, choice of PERT, perceived effectiveness of titration, and the time taken for effective titration. Results: The study comprised 111 participants, predominantly female (93%) and hailing from North America (79%). Of these, 36.7% had been diagnosed with EPI for 3 or more years. A significant 72% felt they were not consistently consuming adequate enzymes, with only 22% believing their intake was sufficient. There were 44 participants (42%) still in the process of adjusting their enzyme doses. In contrast, 17 participants (16%) took a few weeks, 21 (20%) a few months, 11 (10%) over six months, 10 (9%) more than a year, and 3 (3%) several years for dose adjustment. Regarding enzyme titration advice, 30 participants (29%) received vague guidance, while 22 (21%) found the advice beneficial. Conclusions: This study underscores the pressing need for enhanced PERT dosing guidance. The insights gleaned spotlight the prevalent undertreatment across the entire EPI demographic, including in those with lesser-studied co-conditions.

## 1. Introduction

Exocrine pancreatic insufficiency (EPI) can occur from pancreatic diseases, nonpancreatic diseases, or gastrointestinal and pancreatic surgical resection [1]. In certain associated conditions, the prevalence of EPI is estimated to be high. For example, a 2019 review [2] summarizes EPI prevalence estimates, including 30–90% chronic pancreatitis; 80–90% pancreatic duodenectomy; 20–50% distal pancreatectomy; 30–60% benign pancreatic tumors before surgery; 80–90% cystic fibrosis; 20–30% type 2 and 30–50% type 1 diabetes; 15–30% age > 80 years; and 10–20% with tobacco use. A study of older adults in the general population found that the prevalence of exocrine pancreatic insufficiency (EPI) was as high as 11.5%, concluding that there was a clear increase in the prevalence of EPI with age [3]. These data suggest that the diagnosis of EPI is currently suboptimal [4] and that there are many areas for improvement in the understanding of the disease more broadly than the disease-specific research that is currently most represented in the literature.

Since EPI is commonly undiagnosed, even in known high-risk conditions [2], and is routinely undertreated [5,6], it can result in a short-term quality of life impact and can increase morbidity related to osteoporosis and osteopenia [7]. The undertreatment of EPI may be influenced by varied recommendations regarding the optimal dosing of pancrelipase, which is used in pancreatic enzyme replacement therapy (PERT), as well as the myriad formulations available at different strengths [8]. Clinicians and patients may struggle with optimizing PERT for clinical response, patient-reported outcomes, and cost—with little guidance. 

The significance of conducting this survey stems from the need to understand the real-world experiences of individuals with EPI, especially in the context of PERT dosing and how long it takes to arrive at optimal dosing of PERT. The time it takes to achieve optimal dosing, and the ideal approach in order to titrate PERT dosing for individuals, has not been studied. While the current literature offers insights into a few specific co- conditions associated with EPI, there are a palpable lack of comprehensive patient-centric data that reflect the challenges and experiences of those living with the condition outside of relatively rare co-conditions. Many studies with EPI are only designed for specific sub-conditions, and little cross-condition knowledge about EPI is shared in the traditional medical literature. One place such cross-condition knowledge and experiences are shared is in online communities where people living with EPI gather. This study, therefore, sought to understand the real-world experiences of people living with EPI and their use of PERT through an online survey, aiming to identify gaps in current treatment practices, challenges faced by patients, and areas for improvement in patient education and support. By gathering and analyzing these data, we aim to provide momentum to design future studies to support people living with EPI, such as studies on specific approaches to PERT titration. With a deeper understanding of the lived experiences of people living with EPI, we aim to pave the way for more individualized, informed, and effective treatment. 

## 2. Methods

### 2.1. Survey Design

The survey was developed using Google Forms [Alphabet, Mountain View]. The survey could be completed by anyone without requiring registration or submitting an email address. No questions were mandatory and no identifying information was collected. The top of the survey included a description of the study, the author’s contact information, information about what would happen with the survey data, and a reminder that the survey was not required and that any question could be skipped for any reason.

The first section of the survey included demographic questions about age group, geographic region, gender, duration of EPI, and any associated conditions. The second section of the survey focused in more detail on the use of medication to manage EPI and included self-reported elastase levels, whether participants were taking any enzymes for EPI, the type of enzyme, dosage per meal and per snack, typical use patterns, and whether those enzymes were prescription, over-the-counter (OTC), or a combination of both. Participants who were not taking enzymes could articulate their reasons for such in the blanks provided in the survey form. Those taking prescription enzymes were asked about whether they had health insurance and their perceptions of the cost of prescription enzymes. Those taking OTC enzymes were asked about the cost without insurance. Participants were also asked how they decided their dosage of enzymes to take, and if they had a doctor or healthcare provider recommend enzyme treatment; they were provided with an open-ended question for what they thought about the advice or guidance given. Similarly, they were asked if they believed they were taking the ideal dose of enzymes and how long it had taken to arrive at the ideal amount of enzymes (if applicable). Participants were asked whether the cost of enzymes played a role in deciding how much enzyme to take or what to eat; and, similarly, whether they ever changed what they ate in order to change the amount of enzymes that they take. Finally, the survey closed with open-ended questions regarding what they wish HCPs knew about living with EPI; what types of research studies they would like to see related to EPI; what other questions they thought should have been asked in the survey; how they found out about the survey; and anything else they thought should be known about their experiences with EPI.

### 2.2. Patient and Public Involvement

An author (DL) of this study is a patient who designed the study with additional feedback from patients and other community members. An administrator of the Facebook group “Living with Exocrine Pancreatic Insufficiency” was contacted via Facebook Messenger to solicit permission from the group of administrators to post the survey within the group. An initial description of the survey and study design was provided, along with the survey, to the administrator group, who provided feedback and consented for the survey to be posted to the Facebook group. 

### 2.3. Data Collection

The survey link was posted online beginning on 8 March 2022 and the survey closed after three weeks.

### 2.4. Statistical Analysis

The demographics of the study participants, including their age, geographical location, gender, duration of EPI, and any associated conditions, were initially analyzed using the “Summary” tab in Google Forms. The data were then opened in Google Sheets and cleaned. The cleaned survey responses from Google Forms were exported as a CSV file for quantitative analysis using statistical and data science methods. Descriptive statistics, including measures of central tendency, dispersion, and skewness, were calculated for each survey question with non-open-ended responses. 

Google Form’s Responses “Summary” feature also summarized the quantity and percentage of most other numerical statistics reported within the paper. Pivot tables in Google Sheets were used to assess correlations between variables, such as the type of enzymes taken and the average reported elastase levels, the age or duration of EPI and elastase levels, and the duration of EPI and the average quantity and number of PERT pills taken for a typical meal and snack. The average quantity of lipase per meal and snack was calculated by multiplying the dose size of the pancrelipase pills indicated (measured by units of lipase, e.g., 25,000) by the number of pills reported separately for meal and snack intake. The correlations between lipase intake per meal; age; and duration of EPI were calculated compared with elastase levels. 

## 3. Results

### 3.1. Demographics 

A total of 111 participants completed the survey, the majority of whom were female (93%) and from North America (79%). Participants’ ages ranged from 18 to over 75 years, with the largest group being between 55 and 64 years (27%), followed by 65–74 (23%); 45–54 (21%); 34–44 (16%); 25–34 (6%); 75+ (5%); and 18–24 (2%). The duration of EPI diagnosis varied, with most having been diagnosed within 0–6 months (27%); 1–2 years (25%); 5+ years and 3–5 years (both 18%); or 6 months–1 year (12%). Following North America (79%), most participants were in Europe (including the UK, 14%), Australia (5%), and South Africa (1%). 

Of the participants, 102 (92%) indicated that they had received an official EPI diagnosis, while 5 participants had not been officially diagnosed. 

### 3.2. Comorbidities or Related Conditions 

Participants had the opportunity to indicate other possibly related conditions, and 75 (68%) indicated at least one other condition. Diabetes (of any type) was mentioned by 35% (*n* = 26) reporting additional conditions and overall represented in 23% of all participants. Some had type 2 diabetes (*n* = 15), some type 1 diabetes (*n* = 7), while the remainder indicated less-common types (gestational, type 3c, or another type). Celiac disease was the next most-commonly mentioned condition (*n* = 10, 13% of conditions or 9% overall), followed by chronic pancreatitis (*n* = 8, 11% of conditions or 7% overall) and acute pancreatitis (*n* = 4, 5% of conditions or 4% overall). 

### 3.3. Elastase Levels

Most participants had had their fecal elastase levels tested, although 15 (14%) had not. Of those who had and who reported a specific numbered elastase result (*n* = 76), the average was 92 (SD: ±57). Average elastase levels appeared higher in those reporting taking over-the-counter (OTC) enzymes only (average fecal elastase 156) compared with those taking a mix of both prescription and OTC enzymes (average fecal elastase 101) and those taking prescription enzymes only (average fecal elastase 94). In those aged 18–24, the average elastase was 28; those aged 25–34 averaged 50; those aged 35–44 averaged 89; those aged 45–54 averaged 117; those aged 55–64 averaged 92; those aged 65–74 averaged 102; and those aged 75+ averaged 67. A strong negative correlation between age groups and elastase levels, as evidenced by a Spearman’s rank correlation coefficient of −0.704, suggests that as age increases, elastase levels tend to decrease. Similarly, there is a strong negative correlation between duration of EPI and elastase levels, as evidenced by a Spearman’s rank correlation coefficient of −0.806, indicating that longer durations of EPI are also associated with lower elastase levels. 

**Figure 1:**
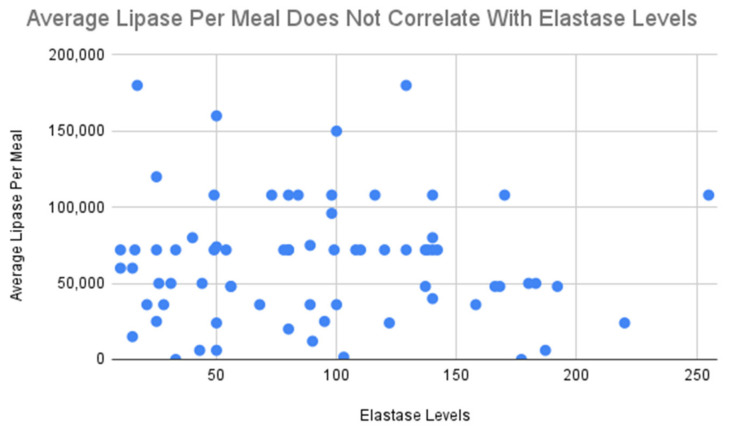
Typical amount of lipase taken for a meal does not strongly correlate with elastase levels in individuals with EPI (*n* = 75).

Elastase levels do not appear to be strongly correlated with the level of lipase units taken per meal (Pearson correlation coefficient: −0.027), as seen in Figure 1. This is similarly true for the amount of lipase units taken per snack (Pearson correlation coefficient: −0.053), which is not strongly correlated with elastase levels.

### 3.4. Enzyme Type and Cost

Most individuals reported taking enzymes (*n* = 100, 90%); of those who did not (*n* = 11), those who reported elastase levels below 200 (*n* = 6, of which *n* = 3 were less than 100) indicate they likely have EPI, but the variety of reasons for not taking enzymes include side effects (*n* = 1); doctor’s advice saying they did not need to take enzymes (*n* = 1); and finding they were not effective (*n* = 1). Only 2 of the 11 participants who reported not taking enzymes indicated that the cost of enzymes played a role in choosing what to take or what to eat. 

Most individuals reported taking prescription enzymes (*n* = 92, 87%), and most of those were Creon (*n* = 75, 80.65%), followed by Zenpep (*n* = 16, 17.20%), and one each reported taking Pancreaze or Viokace. Only five participants reported taking OTC enzymes only, whereas seven individuals indicated they take a combination of prescription and OTC enzymes. 

Of those taking OTC only, Pure Encapsulation (*n* = 3), Vital Nutrients (*n* = 1), and one other unspecified brand (*n* = 1) were mentioned. For reasons to choose OTC enzymes, two individuals indicated side effects with prescription enzymes; two indicated this is what their HCP told them to take; and one indicated cost drove the choice of OTC rather than prescription enzymes. For those taking a combination, three individuals reported taking Creon and Pure Encapsulation; one reported taking Zenpep and Pure Encapsulation; three indicated they were taking Creon and some other brand of OTC. For reasons to choose a mix of OTC and prescription enzymes, four indicated their prescription does not have enough to cover a month and they supplement with OTC, whereas three indicated OTC assists in addressing other symptoms that they have. For those taking OTC alone or in combination, seven (58%) indicated a lack of insurance coverage and that the cost still felt expensive to them. 

For individuals taking prescription enzymes, 37 (40%) had health insurance and reported the cost of prescription enzymes was reasonable, whereas 29 (32%) reported they had health insurance and found the cost of prescription enzymes expensive. Others (*n* = 17, 18%) reported being in varying governmental (federal or state) programs that cover the cost; two indicated they did not have health insurance but have copay or assistance programs covering the cost; one indicated their insurance does not cover PERT. Overall, 71/111 (68%) stated that cost does not play a role in deciding how much enzyme to take or what to eat, while 25/111 (24%) indicated otherwise that cost plays a role. A total of 32% (*n* = 34) of individuals reported they “yes, often” change what they eat in order to change the amount of enzymes they need to take; 20% (*n* = 21) reported “yes, sometimes”, and 40% (*n* = 43) indicated they did not. 

### 3.5. Enzyme Dosing

Similar to elastase levels being correlated with duration of EPI, many participants with longer-duration EPI reported that they do not feel they have their enzymes adjusted or dosed well, which also seems to be emphasized by the varying level of average enzymes per meal and average enzymes per snack taken (shown in Table 1) not correlating with duration of EPI or with elastase levels. There is also a strong negative correlation between the duration of EPI and amount of lipase consumed per meals, with a Spearman’s rank correlation coefficient of −0.801, indicating that longer durations of EPI are typically associated with lower lipase intake per meal.

The majority (*n* = 57, 54%) of people indicated that most of the time, but not always, do they take enough enzymes; only 23 (22%) thought they are always taking enough. Some (*n* = 19, 18%) said they were usually not taking enough enzymes; an additional 11 wrote in comments indicating they were unsure if they were taking enough. 

For determining how much to take, 68 (64%) said the dose their HCP provided worked for them, 27 (26%) said their provider’s instructions was not enough and they had to try taking more, and 13 (12%) indicated they were not told what to take and had to experiment with their dosing.

On average, individuals who reported their elastase levels (*n* = 71) were taking 64,303 (SD: ±39,980) units of lipase per meal (minimum 0; maximum 180,000). There were 14 participants who reported taking less than or equal to 30,000 units of lipase per meal; 7 participants reported taking between 30,000 and 40,000 units of lipase per meal; 6 participants who reported between 40 and 50,000 units of lipase per meal; and 44 participants who reported taking ≥50,000 units of lipase per meal. Table 2 provides quantile and descriptive statistics for total enzyme dosage data per meal and per snack.

For the enzyme dosage data, both lipase per meal and per snack exhibited skewness, with values of 0.769 and 0.873, respectively. Given this skewness, the median and interquartile range (IQR) are particularly informative. For lipase per meal, the median dosage was 72,000 units with an IQR of 38,500 units. For lipase per snack, the median dosage was 36,000 units with an IQR of 14,550 units. While mean and standard deviation are provided for completeness, the median and IQR offer a more robust representation of the central tendency and variability due to the observed skewness.

Regarding the amount of time it took to adjust the ideal amount of enzymes they are taking (see Figure 1 for segmentation by EPI duration), 41.51% (*n* = 44) indicated they have not yet arrived at the ideal amount of enzymes; 16.04% (*n* = 17) suggested it took a few weeks; 19.81% (*n* = 21) took a few months; 10.38% (*n* = 11) took more than 6 months; 9.43% (*n* = 10) took more than a year; and about 2.82% (*n* = 3) took more than a few years. 

## 4. Discussion

Pancreatic enzyme replacement therapy (PERT) is the cornerstone of the management of exocrine pancreatic insufficiency (EPI or PEI). This survey (*n* = 111) offers a comprehensive look into the real-world experiences of individuals with EPI and their experiences with PERT. 

This study is one of the largest surveys available that assesses the real-world experiences of individuals living with EPI, without being limited or targeted to a single associated condition. This study evaluated factors such as age, duration of EPI, elastase levels, choice of pancreatic enzyme replacement therapy (including dose type, size, and amount), perceived titration efficacy, and the time that it took to effectively titrate PERT dosing. 

Most individuals with EPI who responded to the survey do take enzymes, and the majority of those who do take enzymes reported taking prescription pancrelipase alone. Most respondents indicated that they did not think they were taking enough enzymes all the time, which aligns with previous findings that many patients with EPI are undertreated [9,10]. There was not a strong correlation between elastase levels and enzymes taken (−0.027 for meals and −0.053 for snacks). Previous studies have estimated that at least 30,000 IU of lipase per meal is needed [11], whereas a position statement from Italy suggests an initial dose of 40,000–50,000 units of lipase per meal [12], and more recent guidelines also confirm standard practice of 40–50,000 units of lipase per meal [13], whereas other guidelines sometimes vary the starting dose based on associated conditions. While, on average (across *n* = 75 reporting enzyme dose type, size, and quantity), participants were taking 64,303 (SD: 39,980) units of lipase per meal, the dosing varied widely. This combined with the finding that respondents do not think they are taking enough enzymes may surprise clinicians, because the majority (70%) of survey respondents were taking ≥40,000 units of lipase per meal. This may highlight a disconnect between the fact that dosing guidelines typically only state the starting dose range(s) and do not illustrate to readers the real-world dosing that occurs when the guidance is to increase the starting dose by two to three times. For example, the starting dose of 40,000 may increase to 120,000 units of lipase per meal (3×) and the starting dose of 50,000 would increase to 150,000 units of lipase per meal (3×). The skewness score from Table 2 shows the distribution is moderately skewed on the amount of lipase per meal and the amount of lipase per snack is moderately skewed, emphasizing that individual needs vary greatly among the population of individuals with EPI. 

Many people reported that it took a long time to titrate their dosing of PERT (in some cases it took more than a year or a few years, and some do not feel they are well-titrated even after 5+ years of EPI). These are important data that should be followed-up on within additional studies, and in the meantime, providers who support patients with EPI should not assume that duration of EPI is associated with treatment success and improved quality of life. 

Reported levels of elastase are strongly negatively correlated with both age and duration of EPI. This matches additional previous literature studies finding elastase levels correlate with duration of EPI [14,15]. However, the levels of elastase are not correlated (within this population) with levels of enzymes taken and are strongly negatively correlated with duration of EPI. The lack of association found between the level of enzymes taken and any other metric, combined with the amount of time to titrate enzymes, suggests that more should be done around providing advice and guidance to patients with EPI regarding enzyme titration. It is possible that enzyme dosing is well-titrated initially, then dosing is not adjusted over time as elastase levels decrease. Individuals with EPI should be taught a method of PERT dose adjustment that can achieve acceptable symptom management and then be adjusted over time if symptoms recur or gradually worsen. As doses are adjusted, PERT prescription sizes may need to be updated over time. A few of the participants indicated they were taking supplemental OTC enzymes in addition to their prescription because their prescription was not enough to cover an entire month, which supports the hypothesis that prescriptions are not always properly adjusted to match enzyme requirements. Providers should consider regularly assessing with patients whether symptoms are managed and whether the prescription needs updating over time. This study did not assess why participants were taking particular brands of prescription pancrelipase; however, this may reflect the timing in which various options gained regulatory approval [16], and/or lack of awareness that there are six FDA-approved options available, so that if an individual with EPI is not successful with one type of pancrelipase, they could try a different kind. 

Along these lines, dosing advice and guidance should be individualized, as data from this survey show that some with lower elastase are taking relatively small amounts of enzymes relative to standardized guidance. This may occur in individuals who are not aware they could improve titration, or it could be that their symptoms are well-managed at this lower dosing; however, a different individual might have a different outcome with the same or similar levels of elastase and the same enzyme dosing. Like insulin is titrated to the individual based on their bodies’ needs [17,18], so too should PERT be titrated to the individual. One size fits all guidance (e.g., suggesting all patients with moderate or severe EPI need a standard dose and refusing to adjust the prescription accordingly based on patient feedback regarding symptom management) ignores the fact that not only do different people need different levels of enzymes, but that dietary choices vary widely across the patient population. Some individuals with EPI choose to moderate the amount of fat in their diet, despite the literature suggesting that people with EPI need not adjust the fat in their diet [19]. However, most guidance around PERT is based on elastase levels or body weight, and little assessment is given to meal size. Again, like insulin [20], it is possible to titrate enzyme levels based on macronutrients [21] (e.g., fat, protein, and carbohydrate quantity), and this method should be studied further to develop more evidence and to allow for individualized ratios to personalize dosing based on meal quantity to improve outcomes for those who choose to do so. 

Providers should consider what their recommended follow-up plan is for patients newly diagnosed with EPI and ensure clear communication with patients regarding the follow-up plan. If no standard follow-up plan exists in their workflow, they should consider developing a workflow to address this. Similarly, providers who are not the primary managers of EPI care [22] should consider checking in with patients regarding their PERT prescription, titration, and symptom outcomes and supporting them in further titration, even in those patients who present with longer-duration EPI, as this study found around 45% of individuals with EPI for longer than 5 years took a year or more to achieve optimal titration (and over a quarter perceive they are still not optimally titrated). 

Additionally, survey respondents indicated relatively higher rates of diabetes and celiac disease compared with those with pancreatitis in this study. (In the open-ended question, one participant pointed out “not everyone with EPI has pancreatitis”. See examples of other responses to open-ended questions in the Supplementary Materials, including those describing perceptions of healthcare-provider understanding of the lived EPI experience.) While this study is not meant to be a representative sample (see limitations described below) of the entire population with EPI, it is worth noting that many existing studies of EPI are focused within associated conditions, e.g., those with EPI and pancreatitis, and there are fewer estimates of the overall prevalence of EPI in the general population. Previous studies [23] elucidate the profound contribution of PERT to both survival rates and the enhancement of quality of life in patients with EPI. However, this and other studies primarily focus on PERT use in cases of EPI related to cystic fibrosis, pancreatic cancer, and pancreatic-related surgery. These survey data, therefore, add to the body of existing literature suggesting that diabetes [24] and celiac are associated high-risk factors [25] for EPI, and that those living with diabetes and/or celiac disease who present to a gastroenterologist with new-onset GI symptoms warrant EPI screening. The literature establishes this [26], yet awareness among gastroenterologists may be lower for EPI [6] than for other GI conditions such as celiac or gastroparesis with overlapping or similar symptoms in those with diabetes [24]. 

### Limitations

This study is not without limitations, as it was an online survey primarily within those who happen to be members of an EPI-specific Facebook support group, although some respondents also arrived to the survey from Twitter and other social media platforms. Respondents were primarily female, which matches other studies assessing the active membership of online social-media-specific support groups. 

Additional studies should therefore be done to see if these findings further match the experiences of other gender identities, although previous clinical studies with different gender balances (such as another online survey that was predominately (76%) male [27]) also align with these findings. Many participants are seeking to determine if they are achieving the right dosing and whether their remaining levels of symptoms would be considered “normal” for well-titrated dosing or if they are not optimally titrated. This study did not collect data to address this, but future studies should do so, as it shows that patients with EPI desire more guidance around initial and ongoing titration and assessing when one is well-titrated on PERT.

Another limitation of this study is the sample size. While our findings offer a snapshot of the experiences of survey respondents, and this is one of the largest surveys to date in the EPI population without limiting to specific co-conditions, larger-scale studies are desired to validate and generalize these results to the broader EPI community.

## 5. Conclusions

In conclusion, this study is one of the largest online surveys assessing real-world experiences with pancreatic enzyme replacement therapy for individuals living with EPI. Our findings underscore a notable variability in PERT dosing per meal, although many individuals with EPI are still not achieving full symptom resolution. Only one-fifth of people with EPI report perceiving that they are always taking enough enzymes. Many individuals report that it also takes a long time to achieve optimal dosing and many are unsatisfied overall with the advice and guidance they receive about pancreatic enzyme replacement therapy. Many individuals are using over-the-counter enzymes rather than prescription-based PERT, and cost often influences this choice, although cost is not usually reflected in the per-meal dosing decision. Participants perceive that there are gaps in their healthcare providers’ understanding about the impacts of ongoing symptoms of EPI on quality of life, as well as optimal PERT dosing. While elastase levels were correlated with duration of EPI as well as age, the amount of enzymes individuals are taking is not correlated with their elastase levels. However, the duration of EPI was correlated with the amount of lipase consumed. Additional guidance and algorithms to improve individuals’ PERT titration should be developed, and further research should be done to address the relatively high burden of symptoms and overall quality of life impacts of living with EPI without limiting this research to only rare co-conditions.

## Figures and Tables

**Table 1:** Elastase levels, and enzyme amount per meal and per snack segmented by EPI duration. Abbreviations: Avg = average, SD = standard deviation.

Time Since Diagnosis	Count	Elastase (Avg ± SD)	Taking Enzymes	Enzymes per Meal Measured by Units of Lipase (Avg ± SD)	Pills per Meal (Avg ± SD)	Enzymes per Snack Measured by Units of Lipase (Avg ± SD)	Pills per Snack (Avg ± SD)
0–6 months	29	119 ± 52	28	69,243 ± 43,854	2.3 ± 1.1	35,182 ± 25,271	1.1 ± 0.6
6 months–1 year	13	102 ± 68	12	62,192 ± 42,877	2.2 ± 1.0	39,923 ± 29,082	1.2 ± 0.7
1–2 years	27	88 ± 63	24	61,870 ± 40,345	2.2 ± 1.2	34,173 ± 25,957	1.2 ± 0.8
3–5 years	20	76 ± 46	18	57,870 ± 39,709	2.9 ± 1.3	34,173 ± 25,957	1.3 ± 0.9
5+ years	20	47 ± 25	17	65,4875 ± 47,381	2.5 ± 1.7	32,405 ± 21,609	1.2 ± 0.8

**Table 2:** Descriptive statistics for enzyme dosage data.

	Lipase per Meal	Lipase per Snack
Minimum	0	0
Maximum	180,000	108,000
Range	180,000	108,000
Interquartile range (IQR)	38,500	14,500
Standard deviation (SD)	39,979.94	24,192.56
Coefficient of variation (CV)	0.621	0.695
Mean (Rounded)	64,303	34,906
Median absolute deviation (MAD)	24,000	12,000
Skewness	0.769	0.873

## Data Availability

Survey data are available upon request for other researchers. Contact dana@openaps.org for access.

## Supplementary Materials

The following supporting information can be downloaded at: https://www.mdpi.com/article/10.3390/healthcare11162316/s1.
