# Supplementary material for: Survey of Pancreatic Enzyme Replacement Therapy Dosing Experiences in Adults with Exocrine Pancreatic Insufficiency
Source: Healthcare (Basel). 2023 Aug 17;11(16):2316. doi: 10.3390/healthcare11162316 (PMC10454644; doi:10.3390/healthcare11162316)
Supplement: Supplementary file 1 [file healthcare-11-02316-s001.zip › healthcare-2520941-supplementary.pdf]

Supplementary Materials:
Example Responses to Open-Ended Survey Items
About Experiences With EPI and PERT Enzyme Dosing

**Question about enzyme advice from HCPs**	“It was insufficient and unclear. I had to go online and read up myself to understand dosing requirements. I am still figuring out dosing.”
	“Eventually, I increased the amount of Creon I took without my dr approval and was able to start eating more than a few bites. I would have liked a more aggressive approach to finding my dose. Why do I need to suffer longer?”
	“I have just begun this process, however I feel very supported by my GI. I also understand this is going to take a lot of trial and error with diet, enzymes, etc. before I find what is most helpful to me!”
	“It was vague. I was told to take fixed amounts of pills and 'more' for 'big' meals. Whatever that means... I learned online how to calculate number of pills needed according to fat content of the meal and that works better.”
	“Take one pill with meals and snacks and I was prescribed 30 pills. Their initial dosage isn’t working but no info was given about increasing dosage”
**Question regarding what providers should know about living with EPI**	“They should know that PERT doesn’t solve this condition. That there are so many challenges to figure how to deal with it. It is a daily struggle!”
	“They should know that even when we take our prescriptions diligently as ordered, we still can have days of pain and our collective symptoms.”
	“It’s like living with Russian roulette, every day can be different depending what you eat. It affects you mentally as well as physically. It’s exhausting.”
	“They certainly need to educate themselves better from a clinical aspect but they need more understanding of the challenges posed on a daily basis in living with it.”
	“Our bodies are ever changing and so should our prescriptions.”
	“That the symptoms are much more than just words. The "words" aren't strong enough to relate how bad the symptoms really are, i.e. stomach rubbles are more than just a soft noise, they are loud and actually painful”
	“It is a huge mental and emotional upheaval and can be an ongoing burden.”
